# *MiR-146a* reduces fibrosis after glaucoma filtration surgery in rats

**DOI:** 10.1186/s12967-024-05170-2

**Published:** 2024-05-08

**Authors:** Ruiqi Han, Huimin Zhong, Yang Zhang, Huan Yu, Yumeng Zhang, Shouyue Huang, Zijian Yang, Yisheng Zhong

**Affiliations:** 1 Department of Ophthalmology, Ruijin Hospital Affiliated Medical School, Shanghai Jiaotong University, 197 Ruijin Er Road, Shanghai, 200025 China; 2 Department of Ophthalmology, Shanghai General Hospital (Shanghai First People’s Hospital), Shanghai Jiao Tong University School of Medicine, Shanghai, 200080 China; 3 Department of Ophthalmology, Wuxi Branch of Ruijin Hospital Affiliated Medical School, Shanghai Jiaotong University, 197 Zhixian Road, Wuxi, China

**Keywords:** Fibrosis, Glaucoma, Glaucoma filtration surgery, *miR-146a*, Rat Tenon’s fibroblasts, TGF

## Abstract

**Purpose:**

To explore the impact of microRNA 146a (miR-146a) and the underlying mechanisms in profibrotic changes following glaucoma filtering surgery (GFS) in rats and stimulation by transforming growth factor (TGF)-β1 in rat Tenon’s capsule fibroblasts.

**Methods:**

Cultured rat Tenon’s capsule fibroblasts were treated with TGF-β1 and analyzed with microarrays for mRNA profiling to validate miR-146a as the target. The Tenon’s capsule fibroblasts were then respectively treated with lentivirus-mediated transfection of miR-146a mimic or inhibitor following TGF-β1 stimulation in vitro, while GFS was performed in rat eyes with respective intraoperative administration of miR-146a, mitomycin C (MMC), or 5-fluorouracil (5-FU) in vivo. Profibrotic genes expression levels (fibronectin, collagen Iα, NF-KB, IL-1β, TNF-α, SMAD4, and α-smooth muscle actin) were determined through qPCR, Western blotting, immunofluorescence staining and/or histochemical analysis in vitro and in vivo. SMAD4 targeting siRNA was further used to treat the fibroblasts in combination with miR-146a intervention to confirm its role in underlying mechanisms.

**Results:**

Upregulation of miR-146a reduced the proliferation rate and profibrotic changes of rat Tenon’s capsule fibroblasts induced by TGF-β1 in vitro, and mitigated subconjunctival fibrosis to extend filtering blebs survival after GFS in vivo, where miR-146a decreased expression levels of NF-KB-SMAD4-related genes, such as fibronectin, collagen Iα, NF-KB, IL-1β, TNF-α, SMAD4, and α-smooth muscle actin(α-SMA). Additionally, SMAD4 is a key target gene in the process of miR-146a inhibiting fibrosis.

**Conclusions:**

MiR-146a effectively reduced TGF-β1-induced fibrosis in rat Tenon’s capsule fibroblasts in vitro and in vivo, potentially through the NF-KB-SMAD4 signaling pathway. MiR-146a shows promise as a novel therapeutic target for preventing fibrosis and improving the success rate of GFS.

## Introduction

Glaucoma filtration surgery (GFS) plays a crucial role in the treatment of glaucoma by reducing intraocular pressure (IOP) via redirecting the flow of aqueous humor towards the subconjunctival area, resulting in the formation of a bleb. The main challenge of this surgical procedure is scarring of the filtering bleb within the subconjunctival region, which reduces treatment effectiveness. Although extensive prospective randomized studies have demonstrated the efficacy of mitomycin C (MMC) or 5-fluorouracil (5-FU) in preventing scarring and improving GFS outcomes, their clinical use is limited owing to potential risks to vision [1–3]. Therefore, there is an urgent need for innovative antifibrotic medications that can effectively prevent post-surgery scarring.

Transforming growth factor beta (TGF-β) is a cytokine that is crucial for wound healing [4]. TGF-β stimulates transformation of fibroblasts into myofibroblasts, which are primarily responsible for producing fibrous tissues [5]. The formation of scars at the sclerostomy site or bleb connective tissue negatively affects the outcome of filtration surgery because of the abnormal extracellular matrix production and excessive proliferation [6, 7].

Tissue fibrosis stems from the accumulation of the extracellular matrix linked to the persistence of myofibroblasts [8]. Activated rat Tenon’s fibroblasts generate collagen, and TGF-β [5] regulates this process in an autocrine loop by activating TGF-β receptors [9]. Consequently, reduction of collagen expression and suppressing the activation of Tenon’s fibroblasts are crucial for developing treatments for postsurgical scarring. Several studies have recently highlighted the vital roles that microRNAs (miRNAs) play in organ fibrosis [10], proposing a novel approach for regulating fibrotic processes [11]. By base pairing with corresponding 3′-untranslated regions, these small, 22-nucleotide RNAs, known as miRNAs, modify the expression of genes by preventing translation or promoting miRNA degradation. In addition to controlling cell division, apoptosis, and proliferation, miRNAs participate in the developmental and metabolic processes [10, 11]. We employed miRNA microarray analysis to identify the miRNAs involved in bleb scarring and found that nine miRNAs were upregulated in activated rat Tenon’s fibroblasts, whereas seven miRNAs were downregulated. Among these miRNAs, *miR-146a* was significantly up-regulated in TGF-β1-activated rat Tenon’s fibroblasts. Previous studies have shown that *miR-146a* specifically targets a group of mRNAs encoding profibrotic proteins in various tissues, showing promising effects in treating fibrotic conditions in the kidneys [12, 13], liver [14], heart [15], and other organs. Notably, *Smad4* was identified as one of the *miR-146a* target genes [16–18]. Furthermore, *Smad4* deletion attenuated the progression of renal fibrosis by preventing the development of the collagen matrix in obstructive nephropathy and inhibiting the production of collagen I by fibroblasts induced by TGF-β1 [19].

TGF-β activates SMAD4, which then transmits signals to regulate downstream physiological processes. Disruption of *Smad4* decreased renal *Smad7* mRNA expression, promoted renal inflammation dependent on NF-κB signaling, and attenuated the suppressive influence of TGF-β1 on macrophages in vivo and in vitro during an inflammatory response induced by interleukin-1β [20]. Collectively, these findings suggest that SMAD4 could play a substantial role in mediating numerous functions of TGF-β1 in fibrogenesis and inflammation [20].

Postsurgical scarring is an irreversible condition with therapeutic challenges similar to those of organ fibrosis. Identifying concomitant changes in miRNA expression levels suggests the possibility of shared antifibrotic strategies. Postoperative scar development can be treated using miRNA therapy, which effectively treats fibrosis in other organs. Based on previous data about functional significance of *miR-146a* in the fibrosis of various organs and its involvement in the SMAD4 pathway regulation, we postulated that *miR-146a* is associated with the growth of rat Tenon’s fibroblasts and fibrosis resistance. Guided by relevant bioinformatics data and miRNA expression profiles, we investigated the effects of *miR-146a* on the biological features of rat Tenon’s fibroblasts and potential mechanisms of its antifibrotic action. Currently, the role of *miR-146a* in rat Tenon’s fibroblasts remains unknown. Our findings enrich understanding of the properties of rat Tenon’s fibroblasts and offer a novel approach for treating bleb scarring following GFS.

## Materials and methods

### Cell cultures and chemicals

Two 5-week-old SD rats were selected for weighing purposes. Intraperitoneal administration of chloral hydrate 10% anesthesia (0.35 mL/100 g) was performed. The eye area was then treated with proparacaine hydrochloride, a local anesthetic, followed by disinfection using iodine volt. Under the microscope, the subconjunctival Tenon’s capsule tissue was excised. After immersing the tissue in a sterile phosphate buffered solution (PBS) containing double antibodies (100IU/ml Penicillin-Streptomycin) for 30 min, it was transferred to an ultra-clean platform. The tissue underwent two rounds of cleansing with PBS and was subsequently sliced into 1–2 mm implants using ophthalmic scissors. Finally, the implants were arranged in a sterile petri dish measuring 6 cm with a gap ranging from 0.5 to 1.5 cm between them. Following a ten-minute interval, each implant received a droplet of Dulbecco’s modified eagle media (DMEM) and incubated at 37 °C with 5% CO2 overnight. On the following day, Dulbecco’s modified eagle media (DMEM) supplemented with 10% fetal bovine serum (FBS) was added at a volume of 4mL per dish. By replacing the cell medium every 3–4 days, it is possible for the inoculated cells to reach maximum capacity within a culture dish within a period of approximately10 to14 days.

### Rat model of GFS

This trial was approved by the Ethics Committee of the Ruijin Hospital in Shanghai. The Association for Research in Vision and Ophthalmology (ARVO) guidelines were followed for conducting all the experiments. Zhejiang Vital River Laboratory Animal Technology Co., Ltd. (Zhejiang, China) supplied mature male SD rats that weighed around 250 g. The animals were maintained under a 12-h light/dark cycle. Initially, rats received intraperitoneal injections of xylazine (10 mg/kg; Sigma–Aldrich, St. Louis, MO) and ketamine hydrochloride (25 mg/kg; Sigma–Aldrich) to induce a basic sedation state. A drop of 0.5% proparacaine hydrochloride (Tianlong, Suzhou, China) was applied for anesthetizing the eyes being operated on. The left eyes of the rats underwent GFS, as previously reported by Sherwood et al. 2004 [21]. A conjunctival flap, generated by conjunctival incision and a straightforward dissection of the underlying Tenon’s capsule, was positioned 3–5 mm behind the limbus. Subsequently, a 25-G needle was carefully inserted into the anterior chamber to prevent puncture of the iridal blood vessels and to build a full-thickness scleral tunnel. Rats that had any hyphae were excluded. Viscoelastic fluid was injected into the needle to uphold the anterior chamber. Subsequently, a beveled 30-G micro cannula (Qiu Jin, Shanghai, China) was inserted via the scleral tunnel. After securing the microcannula with limbus fixation, the Tenon’s capsule and conjunctiva were sealed with a monofilament nylon suture (10 − 0, 0.1 metric). The animals with eyes that showed cannula slippage or dislocation were excluded. Rats were anesthetized by inhalation of isoflurane (2–4%; Sigma-Aldrich) prior to IOP measurements. 5-FU, MMC (Kyowa Hakko Kirin Co., Ltd., Shizuoko, Japan), normal saline(NS), *miR-146a* mimics, and *miR-146a* inhibitor were administered individually to rats with surgically repaired eyes. Wet the cellulose sponge with MMC (0.4 mg/mL) and 5-FU(25 mg/mL), respectively. Subsequently, a syringe was used to irrigate the treated region with 2 mL of 0.9% sodium chloride. The untreated fellow eyes were maintained under controlled conditions. A minimum of six eyes were included in each group. We used lentiviral vectors (LV) to transfect suitable cells with *miR-146a* according to the manufacturer’s guidelines. GenePharma (Shanghai, China) prepared and identified *miR-146a* LV carrying green fluorescent protein (GFP). Both negative control lentivirus (NC-LV) and recombinant *miR-146a*-LV were generated and subsequently titrated to 1 × 10^9^ transfection units/mL. On postoperative day 1, a subconjunctival injection with a sterile microinjector was performed near the filtering bleb. The expression of *miR-146a* was assessed by routine examination of enucleated ocular tissues. A total of 48 rats (48 eyes) were divided into eight random groups of six rats each with stable IOP (7–20 mm Hg): (1) no surgery—the group without any treatment; (2) sham surgery—this group underwent simple conjunctiva cutting as treatment; (3) MMC—in this positive control group, GFS was performed using a cotton pad with 0.4 mg/mL MMC intraoperatively for approximately 3 min; (4) 5-FU—in this positive control group, GFS was performed using a cotton pad with 25 mg/mL 5-FU intraoperatively for approximately 3 min; (5) surgery + NS—the negative control group was subconjunctivally injected with 25 µL NS on day 1; (6) *miR-146a* mimics—on day 1 following surgery, this experimental group received a subconjunctival injection of *miR-146a* mimics (25 µL); (7) *miR-146a* inhibitor—on day 1 following surgery, this experimental group received a subconjunctival injection of *miR-146a* inhibitor (25 µL); (8) surgery + no tube: This group underwent surgery without the use of a tube, which resulted in the formation of a complete scleral tunnel through the surgical incision of the conjunctiva.

The eyes of the operated rats were closely monitored from postoperative day 1 to day 28 (D1–D28). During this time, we observed and documented the IOP, filtration of blebs, and any subsequent complications. After the surgery, we stained D28 samples with hematoxylin and eosin (HE) and Masson trichrome, as well as using conventional immunohistochemical, immunofluorescent, and real-time PCR approaches.

### Histopathological analysis

All rats were euthanized via forced air embolism following deep general anesthesia and their eyes were excised. The number and dispersion of myofibroblasts and the degree of fibrosis were estimated by Masson staining, HE staining, and immunostaining for α-smooth muscle actin (α-SMA). The primary antibodies were as follows: Vimentin(1:50 dilution; selleckchem, A5862), Cytokeratin(1:50 dilution; selleckchem, A5991), COL1A1 (1:50 dilution; ABclonal, A1352), α-SMA (1:50 dilution; ABclonal, A17910).

### Quantitative RT-PCR

TRIzol (Invitrogen, Wuhan, China) was used to obtain total tissue RNA. Subsequently, a spectrophotometer (Leng Guang, Shanghai, China) was used for quantitative and qualitative analysis of the extracted RNA. We generated cDNAs from 10 ng RNA samples, which were subsequently used for quantitative RT-PCR with the SYBR Green Expression Master Mix (Applied Biosystems, Inc., Foster City, CA, USA). Each experiment was conducted three times. The ΔΔCT technique (2^−ΔΔCt^) was employed to determine the differences in the relative RNA expression levels between the control and treatment groups.

### Western blot analysis

The samples were washed three times with phosphate-buffered saline at 4 ° and then extracted in cold RIPA lysis buffer (strong) composed of 1 mmol/L EDTA, 1% Na_3_VO_4_, 5 µg/mL leupeptin, and 1 mmol/L phenylmethylsulfonyl fluoride, Other components included 2.5 mmol/L sodium pyrophosphate, 150 mmol/L NaCl, 1% Triton X-100, and 20 mmol/L Tris (pH 7.5). The supernatant was centrifuged at 16,099 × *g* for 10 min, quantified, and finally used for western blot analysis.

Immunoreactive proteins were observed on autoradiograph films using chemiluminescence detection reagents (ECL; GE Healthcare, Laurel, MD, USA). Monoclonal antibodies against fibronectin (FN), collagen Iα, α-SMA, SMAD4, NF-κB p65 subunit, IL-1β, and COX2 were obtained from Cell Signaling Technologies and GE Healthcare; β-actin (Sigma-Aldrich Corp., St. Louis, MO, USA) was used as loading control. The primary antibodies were as follows:β-actin (1:1000 dilution; ABclonal, AC026), TNF-α (1:1000 dilution; Abcam, ab205587), IL-1β (1:500 dilution; Abcam, ab205924), α-SMA (1:1000 dilution; ABclonal, A17910), SMAD4(1:1000 dilution; ABclonal, A21487), NF-KB p65 (1:1000 dilution; ABclonal, A2547), Fibronectin (1:1000 dilution; Abways, CY9537), TGFβ1(1:1000 dilution; ABclonal, A2124), COL1A1 (1:1000 dilution; ABclonal, A1352) and COX2(1:1000 dilution; Abways, CY3818).

### Statistical analysis

Each experiment was performed in triplicate. The results are presented as the mean ± standard error of the mean. The Student’s *t*-test was used to statistically analyze the results and compare the differences between the treated and blank groups. Differences between multiple groups were analyzed using one-way analysis of variance. Differences were considered statistically significant if *P* < 0.05.

## Results

### Expression of *miRNA146a* increased in TGF-β1-stimulated rat tenon fibroblasts

**Fig. 1:**
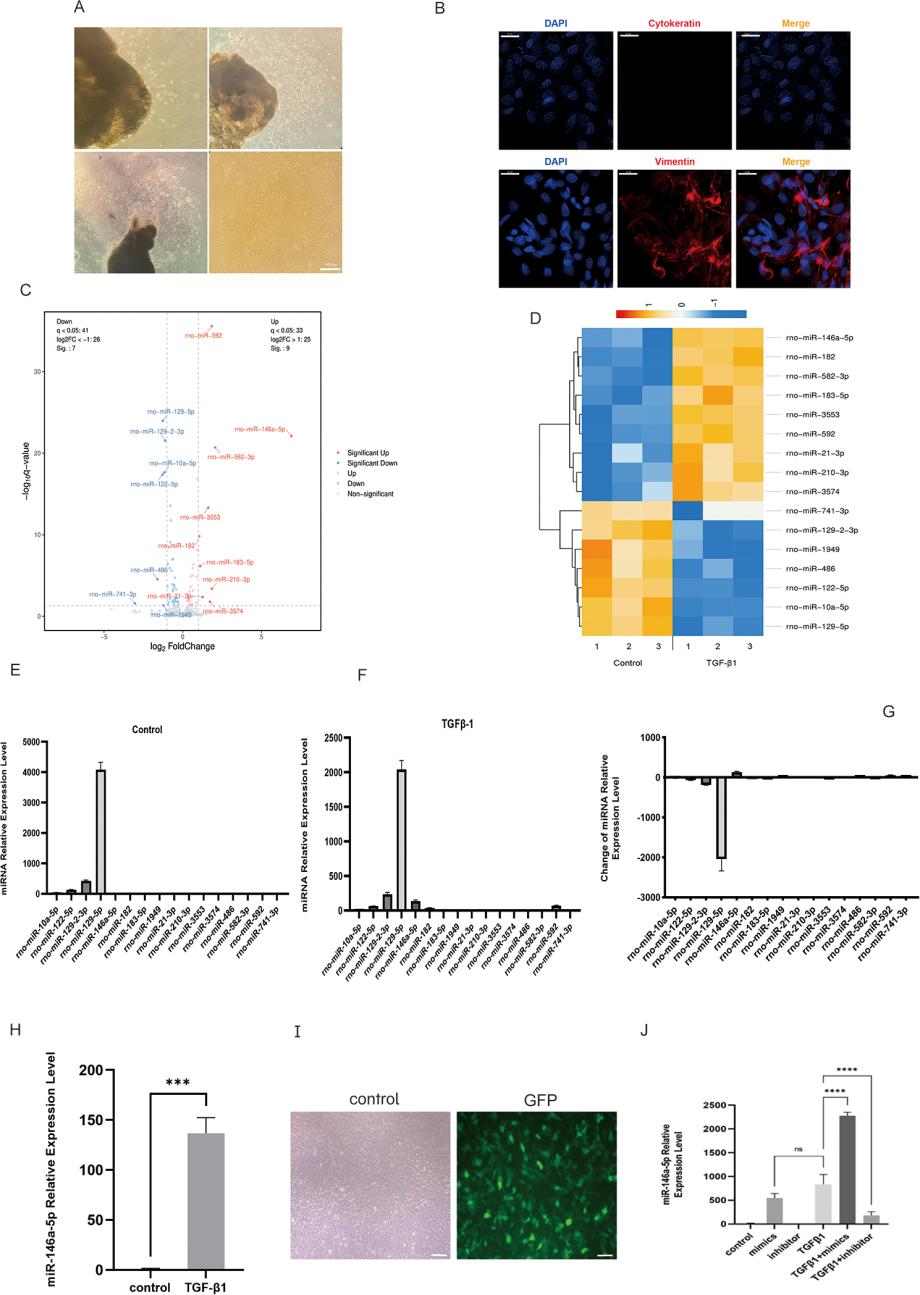
The expression of *miR-146a* is obviously increased in TGF-β1-stimulated rat Tenon’s fibroblasts. After 4–7 days, the cells moved out of the adhering rat Tenon’s tissue, and following passages, achieved confluence of 90% or more. Images were captured at a magnification of 100× (**A**). Staining of rat Tenon’s fibroblasts with antibodies against vimentin, cytokeratin, and fibroblast surface protein. Blue indicates nuclei staining with 4′,6-diamidino-2-phenylindole (DAPI); red indicates vimentin staining; green indicates Cytokeratin staining. Cytokeratin staining is negative. Each image was captured at 400× magnification (**B**). The volcano plot shows differentially expressed miRNAs. Red, blue, and gray indicate up-regulated, down-regulated, and unchanged miRNAs, respectively. Fold changes were used to express the values in relation to the corresponding miRNA levels in quiescent primary rat Tenon’s fibroblasts (**C**). Expression pattern cluster analysis was used to create a heat map representation of miRNAs (**D**). miR microarray analysis demonstrates a substantial (∼ 120-fold) increase in *miR-146a* expression in TGF-β1-stimulated rat Tenon’s fibroblasts compared to that in the untreated fibroblasts (**E-G**). Quantitative real-time RT-PCR reveals a significant increase in *miR-146a* expression (***P < 0.001) in rat Tenon’s fibroblasts stimulated with TGF-β1, thereby confirming microarray data (**H**). After 3 days, the transfection rate of lentivirus reached 90%, as indicated by green fluorescent protein expression. Images were captured at 100× magnification (**I**). Rat Tenon’s fibroblasts treated with *miR-146a* mimics showed a substantial rise in miR-146a expression compared to that in controls or in TGF-β1-treated cells (****P < 0.001). An inhibitor of *miR-146a* had opposite effects. There was no significant difference between group mimics and group TGF -β1 (**J**)

Tenon’s capsule fibroblasts were cultured by explant method. About 4 days after the primary culture of rat Tenon’s capsule fibroblasts, slender spindle-shaped cells could be seen crawling out of the tissue block, and the cells continued to expand from the center of the graft to the surrounding area. After 8–10 days of culture, the degree of confluence of cells could reach about 70%. Under the microscope, the cells showed a spindle-shaped appearance and a vortex arrangement. At 14 to 16 days, the degree of cell confluence could reach 90%, and microscopically, the cells appeared as closely packed monolayers with slightly polygonal changes in appearance (Fig. 1A). Tenon’s capsule fibroblasts were mesenchymal derived cells. Vimentin was a marker of mesenchymal derived cells, and Cytokeratin was a marker of epithelial derived cells. Immunofluorescence staining of Tenon’s capsule fibroblasts showed that the red fascicles or reticular structures in the cytoplasm of Vimentin immunofluorescence staining positive cells were consistent with the direction of the long axis of the cells, and Cytokeratin immunofluorescence staining was negative. The results were consistent with the characteristics of Tenon’s capsule fibroblasts without epithelial cell contamination. It indicated that the purity of the cultured primary rat Tenon’s capsule fibroblasts was high (Fig. 1B).

Next, to identify miRNAs involved in scarring following filtration surgery, rat Tenon’s fibroblasts were treated with TGF-β1, and their miRNA expression profiles were compared to those in the untreated primary rat Tenon’s fibroblasts (control group) from the same passages (Fig. 1C-G). Out of the 507 distinct miRNAs displayed on the microarrays, 16 had significantly changed expression in rat Tenon’s fibroblasts upon stimulation with TGF-β1. Expression levels of nine of these miRNAs were more than 2-fold higher, whereas for seven of them, expression levels were more than 2-fold lower than those in control cells (Fig. 1C, D). Expression pattern cluster analysis was used to create a heat map representation of miRNAs that showed a 2-fold or greater change in expression in activated rat Tenon’s fibroblasts following 24 h stimulation with TGF-β1 (Fig. 1D). miR microarray analysis demonstrates a substantial (∼ 120-fold) increase in miR-146a expression in TGF-β1-stimulated rat Tenon’s fibroblasts compared to that in the untreated fibroblasts (Fig. 1H).

### Administration of *miR-146a* mimics enhances the persistence of filtering blebs following GFS in rats

**Fig. 2:**
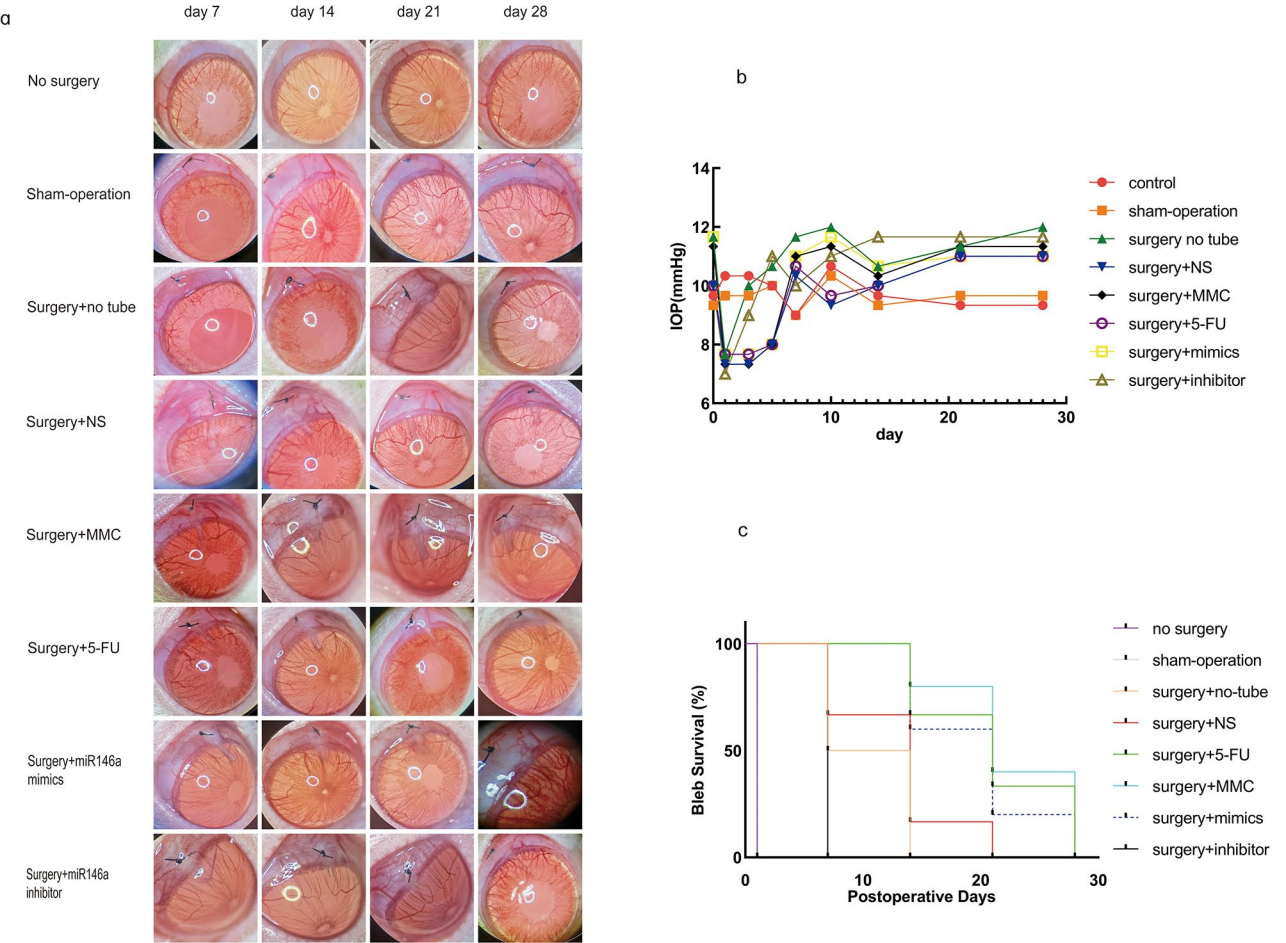
MiR-146a mimics enhanced filtering bleb survival following GFS. Administration of *miR-146a* mimics enhances the persistence of filtering blebs following GFS in rats. Development of filtration blebs in no surgery, sham surgery, MMC, 5-FU, surgery + NS, *miR-146a* mimics, *miR-146a* inhibitor, and surgery + no tube eyes groups at Day7, Day14, Day21, and Day28 (**A**). IOP scores on Day1- Day28 in the no surgery, sham surgery, MMC, 5-FU, surgery + NS, *miR-146a* mimics, *miR-146a* inhibitor, and surgery + no tube groups post GFS (**B**). Persistence of filtering blebs in no surgery, sham surgery, MMC, 5-FU, surgery + NS, *miR-146a* mimics, *miR-146a* inhibitor, and surgery + no tube groups following GFS (**C**)

Following GFS, there were no obvious congestive, cataract and other complications in anterior segment in each group (Fig. 2A) and there was no significant change in IOP between the treatment groups. Consequently, the IOP of these two groups was not much lower than that in the blank group on postoperative Day21 (Fig. 2B) (*P* > 0.05).

A functional filtering bleb is crucial for regulating IOP after GFS. Therefore, we investigated the influence of seven different treatments on the durability of filtering blebs following GFS. The outcomes of the bleb survival analysis revealed a significant effect of treatment on the survival rates in the surgery alone, *miR-146a* mimics, MMC, and 5-FU groups (*P* < 0.001). Post-GFS, rats in the surgical “no tube” group experienced a steep decline in filtering blebs, with a paucity of such blebs observed at Day14. The persistence of filtering blebs in the mimics, MMC, and 5-FU groups was notably greater in comparison to that in the surgery + NS group. The degradation of filtering blebs in the 5-FU group started on Day14 and finished by Day28. In contrast, filtering blebs in the MMC group survived noticeably longer than those in the 5-FU group. The degradation of filtering blebs in the MMC group commenced on Day14, and 50% of the blebs were still present at Day28. Filtering blebs disappeared in the *miR-146a* mimics group on Day21, but 30% of them still persisted at Day28. Compared to the observations in the surgery plus NS group, therapy with *miR-146a* mimics significantly enhanced bleb survival (*P* < 0.001). Altogether, these results indicate that treatment with *miR-146a* mimics enhanced filtering bleb survival following GFS, and its benefits seem to be as substantial as 5-FU (Fig. 2C).

### In vivo transfection of miR-146a regulates fibrosis

**Fig. 3:**
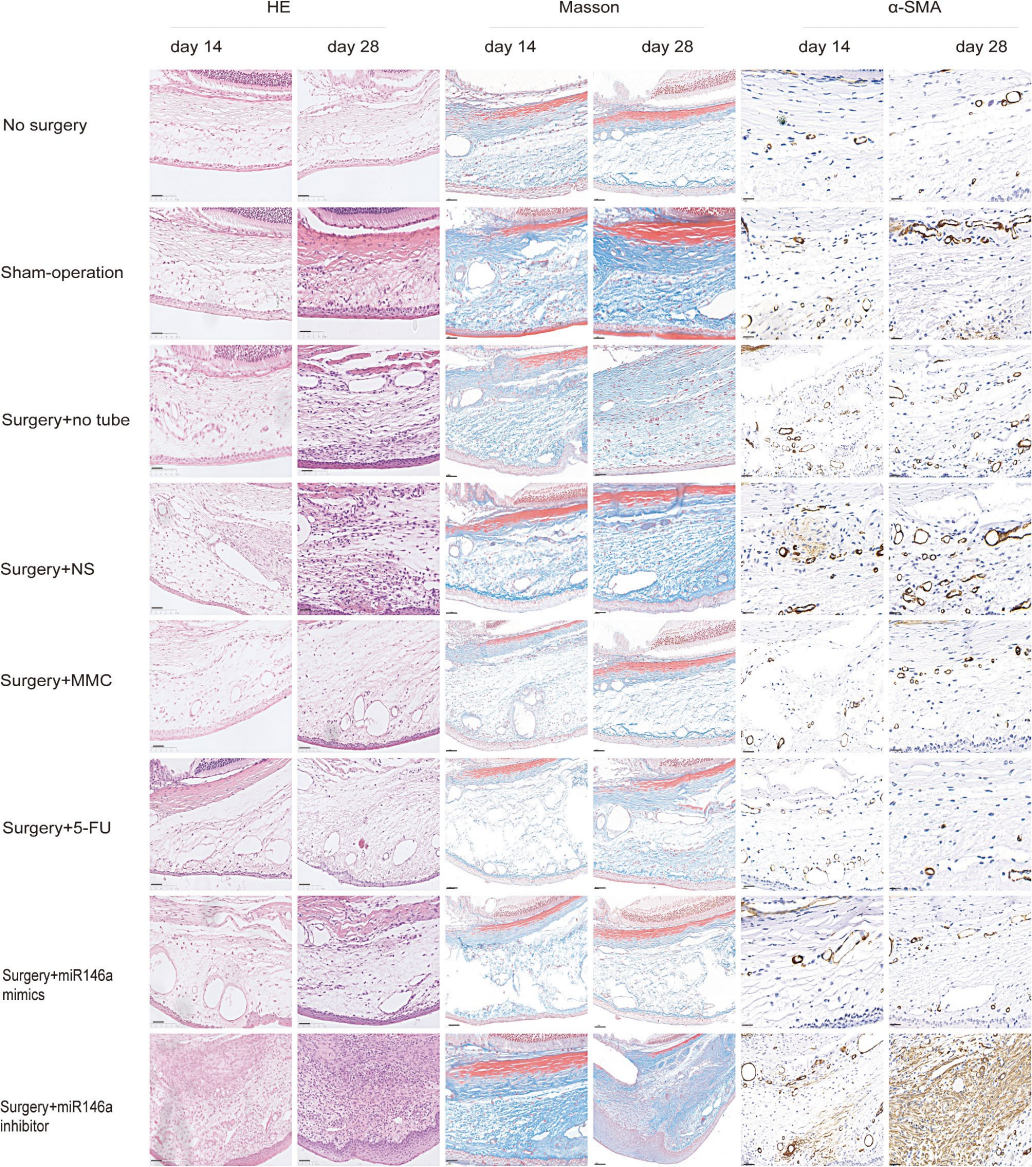
MiR-146a mimics reduced subconjunctival fibrosis after GFS surgery. Analysis of the conjunctiva and sclera within the bleb region of the eight experimental groups using HE, Masson trichrome, and α-SMA immunohistochemistry staining (scale bar, 50 μm)

On day 1 postoperatively, eye operation sites were subjected to a subconjunctival injection of miR-146a-LV (25 µL). Masson and HE staining of samples was performed on postoperative Day14 and Day28. The collagen development between the conjunctiva and sclera of the operated area dramatically reduced in the experimental group transfected with *miR-146a*-mimics, as shown by Masson trichrome staining. We observed some collagen production in the positive control group. Collagen was heavily deposited in the groups that underwent a single surgery and in the negative control group. To ascertain the extent of fibrosis and collagen deposition, we performed α-SMA immunohistochemistry staining. In the blank group, α-SMA was not expressed between the conjunctiva and sclera. However, under the blebs, α-SMA was strongly expressed in the groups that underwent a single surgery and in the negative control group. α-SMA was extensively expressed at the surgery site, but modestly expressed in the positive control group. Moreover, α-SMA expression was infrequent in the experimental group. At Day28, the eyes treated with *miR-146a* had significantly less collagen deposition than the operated eyes in other groups. Based on these findings, we inferred that transfection with *miR-146a* mimics dramatically suppressed the expression of α-SMA mRNA in the rat model of GFS (Fig. 3).

**Fig. 4:**
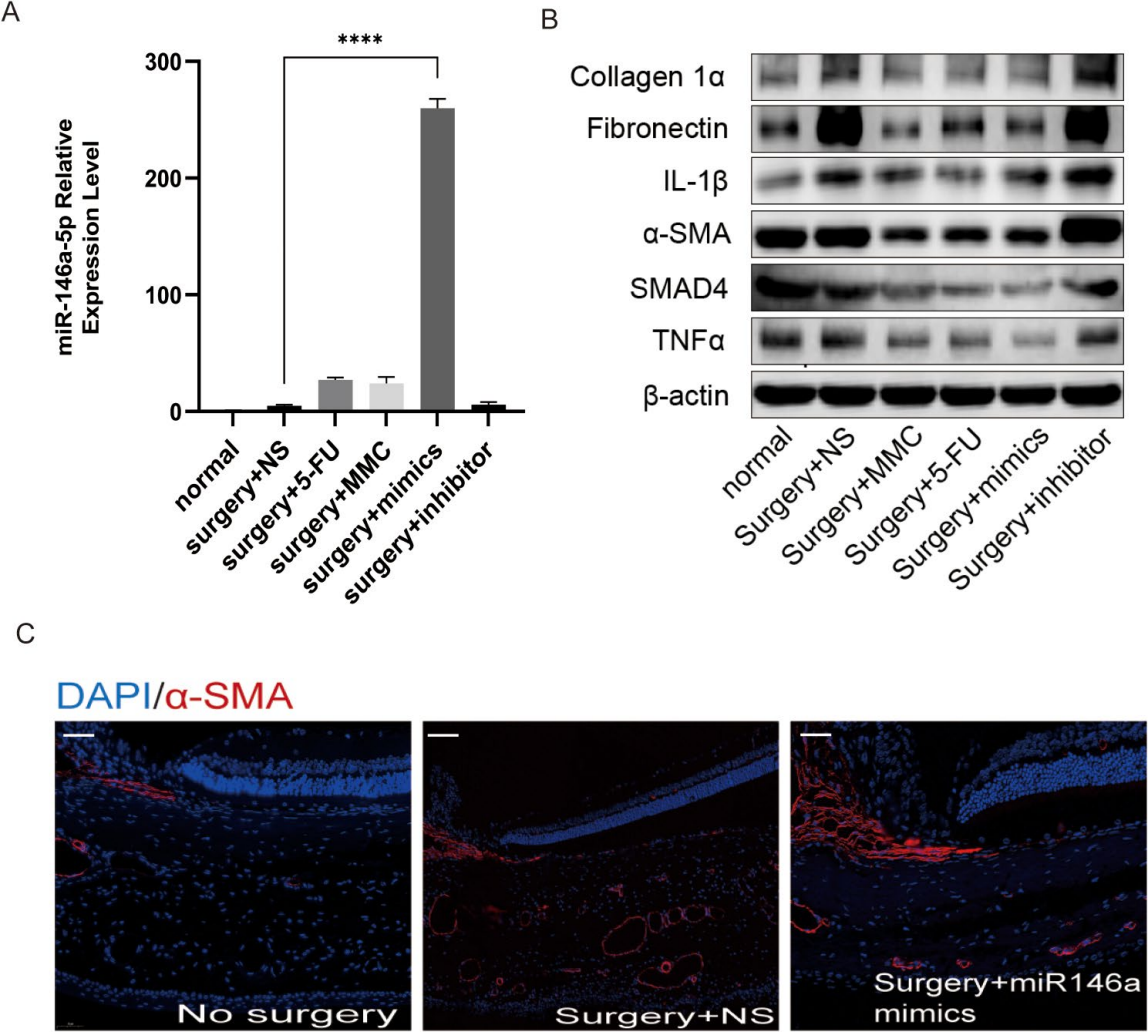
MiR-146a inhibits fibrosis in vivo. Polymerase chain reaction analysis of changes in miR-146a expression after injections of NS, 5-FU, MMC, or transfection with *miR-146a* mimics and inhibitors (**A**). Representative western blot images of α-SMA, FN, TNFα, IL-1β, Col1A1, and SMAD4 protein levels normalized by β-actin levels (**B**). Blue (DAPI) and red staining indicate nuclei and α-SMA expression, respectively (**C**). Bar scale: 100 μm; magnification: ×20

Western blot analysis revealed significant reductions in α-SMA, FN, TNFα, IL-1β, Col1A1, and SMAD4 levels in the surgery + NS, negative control, positive control, experimental, and blank groups. Furthermore, the degree of these reductions positively correlated with *miR-146a* expression (Fig. 4A-C).

### TGF-β1 promotes proliferation of Rat Tenon’s fibroblasts

**Fig. 5:**
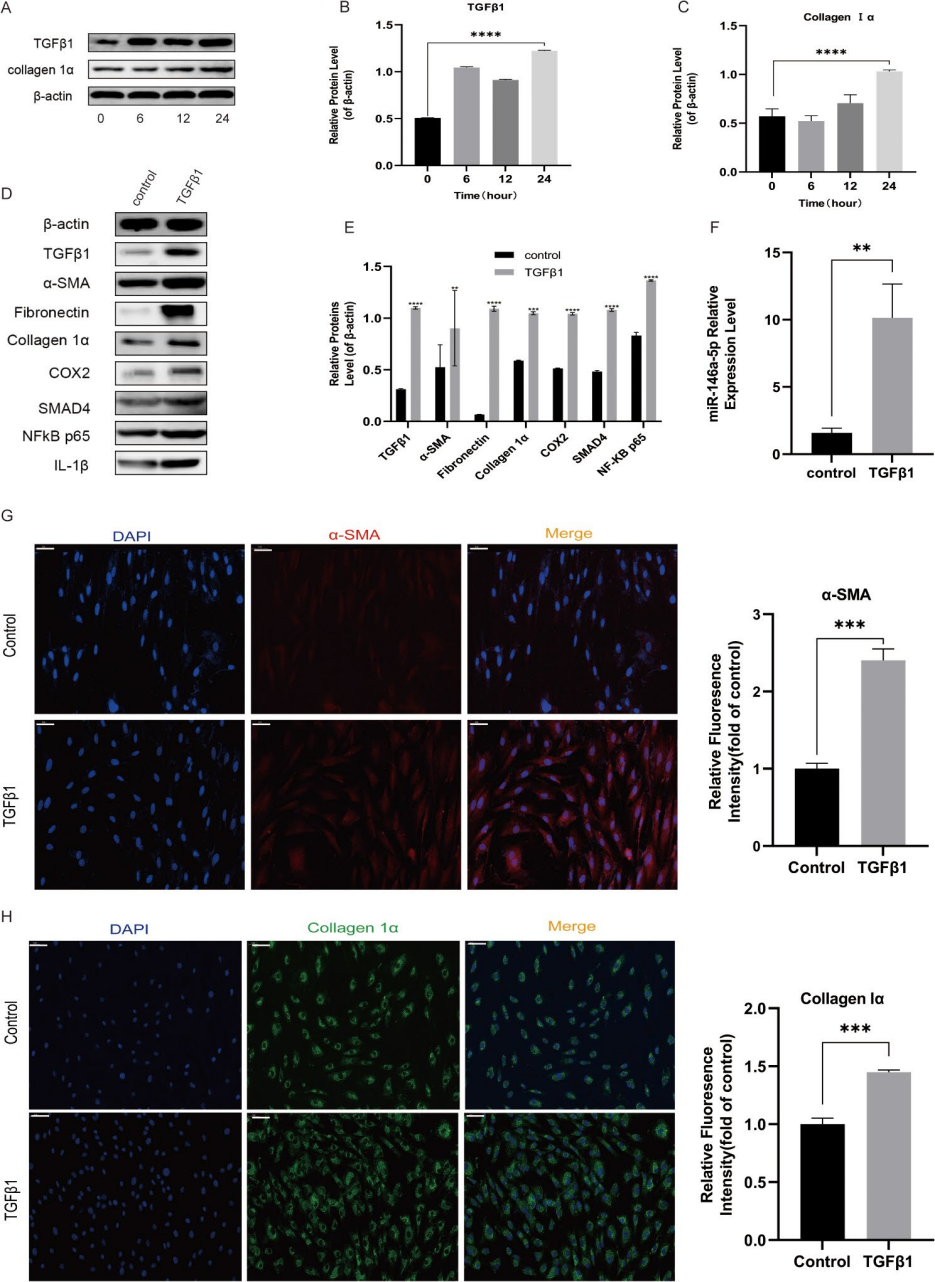
TGF-β1 Promotes Proliferation of Rat Tenon’s Fibroblasts. TGF-β1 stimulates the development of rat Tenon’s fibroblasts. Rat Tenon’s capsule fibroblasts were treated with 10 ng/mL TGF-β1 for 0, 6, 12, or 24 h (**A**). Western blot assays was performed to determine the expression of cell fibrosis markers TGF-β1 and collagen Iα related to cell proliferation. Compared to controls, **** *P* < 0.001 (**A-C**). Treatment of rat Tenon’s fibroblasts with TGF-β1 for 24 h led to a significant rise in *miR-146a* expression compared to that in control (untreated) cells; **P < 0.01 (**F**). Staining of TGF-β1-treated and untreated (control) rat Tenon’s fibroblast cells with antibodies against α-SMA, collagen Iα, and fibronectin protein. Red and blue indicate α-SMA and nuclear staining (**g**), Nuclei were identified with DAPI (blue) and collagen Iα-positive staining (green) (**H**) ***P < 0.001. Every image was captured at 400× magnification. Extended treatment with TGF-β1 led to elevated expression of COX2, SMAD4, NF-κB p65, α-SMA, collagen Iα, TGF-β1, IL-1β, and total FN (**D, E**). The information in the columns presents the average relative density ratio ± standard deviation of three distinct experiments, all adjusted to the β-actin concentration within the same specimen. Statistical significance of differences between treated and untreated cells is illustrated as follows: *P* < 0.05

It has been shown previously that TGF-β1 strongly promotes fibroblast proliferation. To examine its impact on rat Tenon’s fibroblasts, TGF-β1 was applied to these cells at a concentration of 10 ng/mL for 0, 6, 12, or 24 h. The western blot assay findings indicated a time-dependent increase in cell proliferation, which was further supported by the production of two indicators of cell fibrosis, TGF-β1 and collagen Iα (Fig. 5A-C).

We then examined the amounts of FN, collagen Iα, α-SMA, SMAD4, NF-κB p65, IL-1β, and COX2 in the Tenon’s tissues in both normal participants and those treated with TGF-β1. Western blot analysis and immunofluorescence revealed that all the molecules that may activate rat Tenon’s fibroblasts had substantially greater proteins expression levels in TGF-β1-treated patients than in control subjects (Fig. 5D, E,G, H). Moreover, TGF-β1 therapy increased *miR-146a* expression (Fig. 5F).

### Impact of *miR-146a* mimics on rat Tenon’s fibroblasts stimulated with TGF-β1

**Fig. 6:**
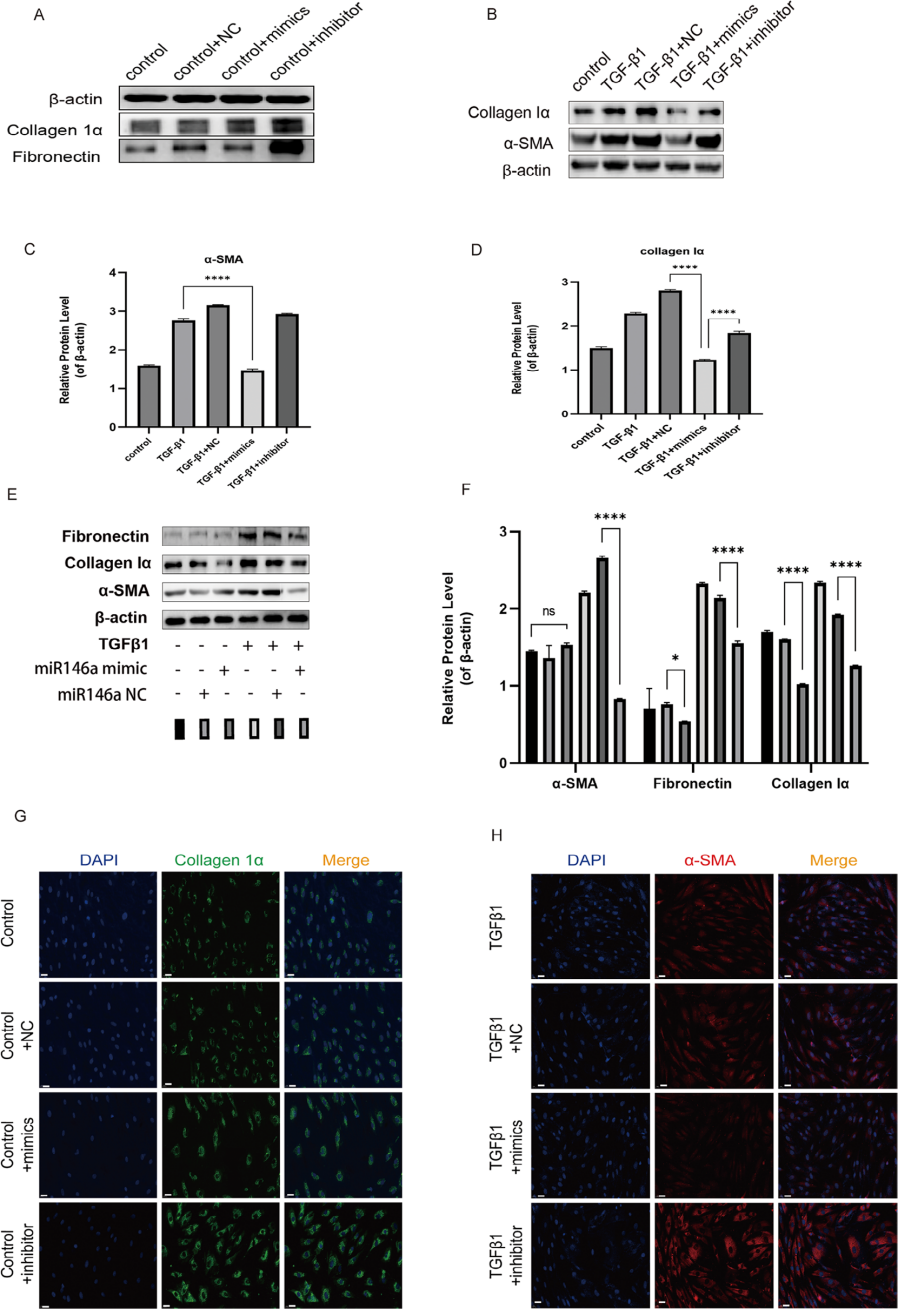
MiR-146a inhibits TGF-β1-induced myofibroblast transdifferentiation. Representative western blot images of α-SMA, FN, Col1A1 protein levels normalized by β-actin levels (**A-F**). Nuclei were stained using 4′,6-diamidino-2-phenylendole (DAPI) and visualized ×20 magnification. Bar scale: 100 μm. Immunofluorescence staining for α-SMA (red) and collagen Iα (green) (×20 magnification. Bar scale: 100 μm) (**G, H**). *miR-146a* mimics and inhibitors, respectively, decreased and increased expression of α-SMA and collagen Iα in control and TGF-β1-treated fibroblasts of rat Tenon’s capsule. The information in the columns shows the average relative density ratio ± standard deviation of three distinct experiments, all adjusted to the β-actin concentration within the same specimen. Statistical significance of differences between treated and untreated cells is shown as *P < 0.05

Using western blotting, we next investigated the impact of *miR-146a* mimics on rat Tenon’s fibroblasts with respect to expression of fibrotic markers FN, collagen Iα, and α-SMA. Stimulation with TGF-β1 augmented expression levels of FN, collagen Iα, and α-SMA, and these increases were reversed by miR-146a mimics (30 nM, Fig. 6A-F).

To examine the role of *miR-146a* in TGF-β1-induced myofibroblast transdifferentiation, rat Tenon’s fibroblasts were treated with miRNA mimics and inhibitors (Fig. 6A-C). Following transfection, inhibitors of *miR-146a* enhanced fibrosis expression, whereas *miR-146a* mimics had an opposite effect (Fig. 6D-H). These findings suggested that TGF-β1-induced myofibroblast transdifferentiation may be impacted via *miR-146a* expression modulation.

### *MiR-146a* regulates fibrosis by targeting SMAD4

**Fig. 7:**
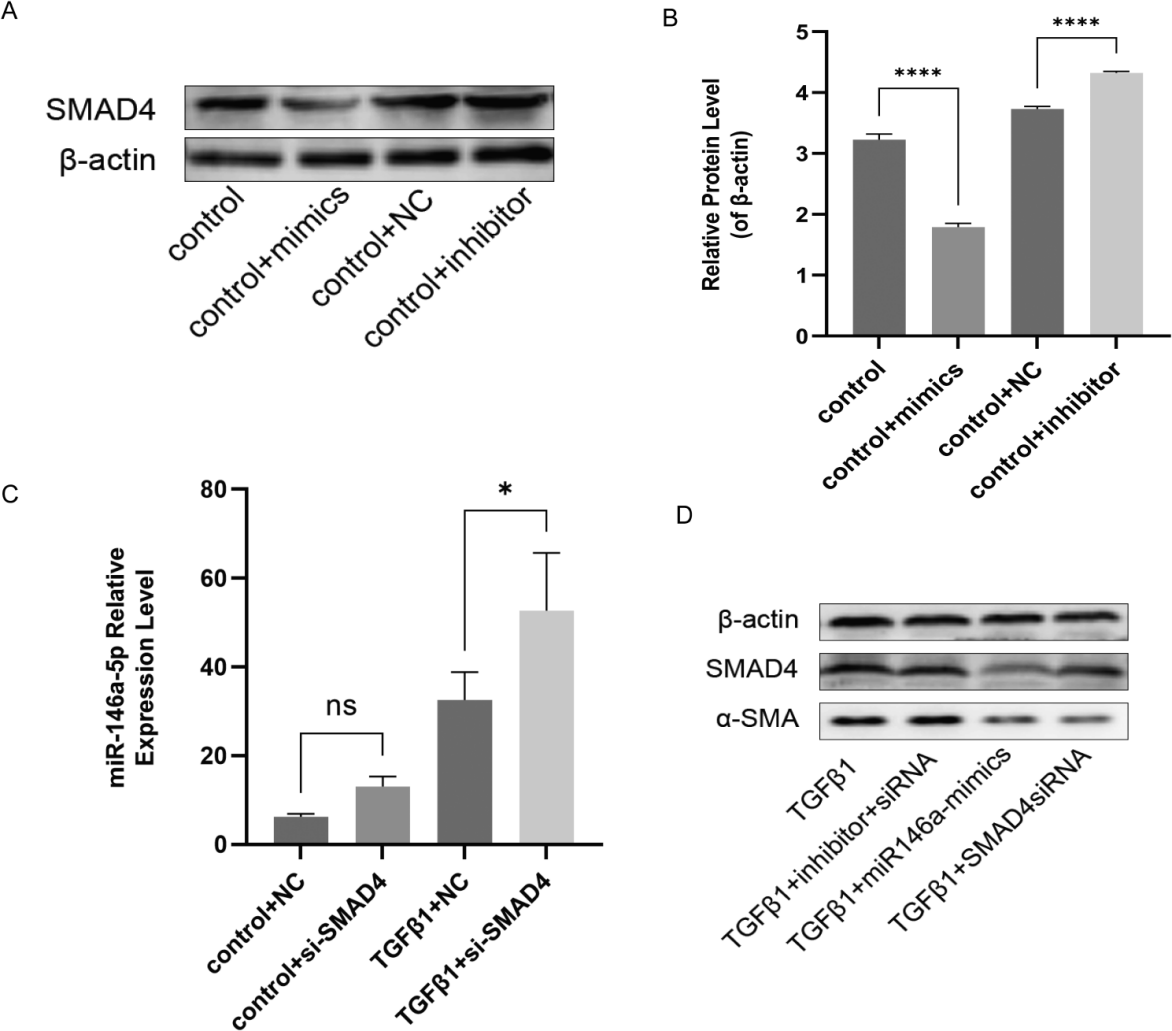
MiR-146a Regulates Fibrosis by Targeting SMAD4. Representative western blot images showing SMAD4 protein level normalized by β-actin protein level (**A, B**). Polymerase chain reaction analysis of changes in miR-146a expression after transfection with siRNA against *SMAD4* (**C**). Representative western blot images showing SMAD4 protein level normalized by β-actin protein level (**D**)

Transfection with a *miR-146a* mimic specifically targets the 3′-untranslated region of *Smad4* mRNAs in various cells. SMAD4 protein level decreased significantly after transfection with *miR-146a* mimics, compared with that in cells treated with control mimics (Fig. 7A, B). Furthermore, SMAD4 expression increased significantly upon *miR-146a* expression inhibition compared with that in the presence of control inhibitors. We also transfected rat Tenon’s fibroblasts with small interfering RNA against *SMAD4* and found that the decreased SMAD4 expression increase *miR-146a* expression (Fig. 7C).

After transfection with miR-146a mimics and SMAD4-siRNA, the levels of both Smad4 and α-SMA proteins were significantly reduced compared to those treated with TGFβ1. Conversely, the expression levels of Smad4 and α-SMA showed a noticeable increase when using miR-146a-inhibitor + SMAD4-siRNA instead of incorporating miR-146a analogs and SMAD4-siRNA (Fig. 7D).

## Discussion

GFS is a surgical intervention typically performed when conservative treatment outcomes are unfavorable. However, most patients develop bleb scarring following GFS, which is the primary cause of surgery failure. Notably, the intra- and postoperative application of chemotherapeutic drugs 5-FU and MMC significantly reduce the occurrence of postoperative bleb scarring. However, the systemic use of these drugs can lead to cellular damage, bleb leakage, consistently low IOP, corneal scarring, scleritis, and endophthalmitis. Therefore, innovative therapeutic strategies are being developed to enhance the success rate of GFS by suppressing scar formation and inhibiting fibroblast proliferation within the subconjunctival tissue. In previous studies, researchers experimented with various biological, chemical and physical approaches to suppress fibroblast proliferation and scar tissue formation, including the inhibition of TGF-β1, vascular endothelial growth factor [22], Rho-associated protein kinase [23], as well as photodynamic therapy [24], and others. Although some of these treatments had remarkable results and improved GFS outcomes in the standard rat model, only few of them were successful in large-scale prospective clinical trials. Thus, so far, none of these strategies have replaced MMC or 5-FU use in clinical practice. MiRNAs are crucial regulators of fibrotic processes, and promising target candidates for treating fibrosis in various organs [25]. Tenon’s fibroblasts, which are the primary fibrotic cells in the Tenon’s tissue, contribute to bleb scarring in rodent models after they transdifferentiate into myofibroblast-like cells. In vitro assessments have revealed that the number of myofibroblasts decrease when *miR-146a* is directly targeted [26].

In the present study, we used LV-facilitated transfection for gene delivery in vivo. We performed RT-PCR and western blot analysis to detect the expression of the transfected genes and assess alterations in their protein levels, respectively. In rats that underwent GFS, *miR-146a* was successfully transfected when LV concentrate was injected into the subconjunctiva adjacent to the filtering bleb. The resulting up-regulation of *miR-146a* expression inhibited the formation of new collagen in the surgical site by suppressing SMAD4 signaling pathway. We propose that *miR-146a* effectively reduces collagen deposition when delivered exogenously, thereby suppressing bleb scarring in rats undergoing GFS. To the best of our knowledge, this is the first time that in vitro and in vivo studies have collectively confirmed that delivering miR-146a can efficiently prevent scarring of the subconjunctival tissue in rats subjected to GFS. Hence, our novel therapeutic approach could be employed in glaucoma surgery in the near future. Previous studies have established various strategies to explore the functional role of *miR-146a* in fibrosis and to identify *miR-146a* target genes [16, 27–29]. However, these studies have primarily employed in vitro experimental designs. For instance, Gordon et al. [15] utilized human and mouse cardiac microvascular endothelial cells to demonstrate that *miR-146a* negatively regulates NF-κB and *Col1α1* mRNA expression. Luna et al. [30] showed that rats treated with *miR-146a* had a sustained reduction in IOP, without any observable signs of inflammation or other adverse effects. Sun et al. [26] found that the expression of FN, collagen Iα, and α-SMA protein induced by TGF-β1 treatment was reduced upon the introduction of miR-146a mimics. Additionally, SMAD4 protein levels were significantly decreased in response to *miR-146a* mimics [26]. Zhang et al. [31] and Kim et al. [32] found that *miR-146a* expression is enhanced in response to various inflammatory stimuli, and anti-inflammatory effects can be achieved by regulating *miR-146a* expression. Based on these results, we developed a rat model of GFS and tested gene therapy to prevent scarring. Usually, these strategies require long-term transgene expression in subconjunctival tissues. We fulfilled this prerequisite condition by using a single subconjunctival injection of a concentrated preparation of LVs harboring *miR-146a* in our rat model. Although 28 days was not sufficient for the experiment to be considered long-term, transfected GFP gene was abundantly expressed and visualized in vivo in the enucleated tissues obtained from the operated sites. Although identifying a therapeutic transgene is challenging, we cannot downplay the clinical advantages of gene therapy in hindering bleb scarring.

This study had several limitations. As previous studies have not performed LV-mediated *miR-146a* transfection into rats that had undergone GFS, we had to determine the transfection duration based on the characteristics of the fibrotic process: after undergoing GFS, fibroblast proliferation increased in rats. The conjunctival wound healing in these rats takes place within 7–14 days post-surgery. Additionally, we were unable to determine the optimal frequency and dosage for subconjunctival injections. Prior studies have shown that in vivo LV-mediated transfection has low efficiency, indicating a need for further evaluation of whether gene therapy is a suitable method. In this study, we used a high titer LV preparation and administered it in the undiluted form to maximize transfection levels. However, factors such as the selection of promoters, quality of vector preparation, and viral dose must be carefully considered in the future studies. Moreover, we presumed that vector particles were not shed in tears or aqueous humor. These assumptions are valid for short observation periods; however, future studies should explore whether time-dependent changes affect transfection efficiency.

## Conclusion

our study is the first one to describe LV-mediated transfection of microRNA for gene delivery in an animal model of GFS. Drawing on our collective research findings, we believe that modulating *miR-146a* levels could mitigate bleb scarring following GFS.

## Data Availability

The datasets used and/or analyzed during the current study are available from the corresponding author on reasonable request.
